# Comparison of emergency pediatric breast ultrasound interpretations and management recommendations between pediatric radiologists and breast imaging radiologists

**DOI:** 10.1007/s10140-022-02081-x

**Published:** 2022-08-16

**Authors:** Derek L. Nguyen, Emily B. Ambinder, Lisa A. Mullen, Eniola T. Oluyemi, Emily A. Dunn

**Affiliations:** The Russell H. Morgan Department of Radiology and Radiological Science, Johns Hopkins University School of Medicine, Baltimore, MD 21287 USA

**Keywords:** Breast ultrasound, Pediatric, emergency department

## Abstract

**Purpose:**

Pediatric patients with breast-related symptoms often initially present to the emergency department for evaluation. While pediatric radiologists are accustomed to evaluating acute infectious and traumatic etiologies, they may be less familiar with breast-specific findings. This study compares management recommendations of pediatric breast ultrasounds performed in the emergency setting between pediatric and breast imaging radiologists.

**Methods:**

This retrospective cohort study reviewed data from all pediatric breast ultrasounds performed in the emergency setting from a single academic institution from 1/1/14 to 12/31/19. During the study period, 12 pediatric radiologists with experience ranging from 1 to 33 years interpreted pediatric breast ultrasounds. Three breast imaging radiologists (with 3, 8, and 25 years of experience) retrospectively reviewed each case and recorded whether further management was recommended. Differences in recommendations were compared using Fisher’s exact test. Cohen’s kappa was used to assess agreement between subspecialty radiologists.

**Results:**

This study included 75 pediatric patients, with mean age 13 ± 5.6 years and malignancy rate of 1.3% (1/75). Pediatric radiologists and the most experienced breast imaging radiologist had moderate agreement in management recommendations (*k* = 0.54). There was no significant difference in recommendations for further management between pediatric radiologists (22/75 [29.3%]) and the most experienced breast imaging radiologist (15/75 [20.0%]), *p* = 0.26.

**Conclusion:**

Recommendations for pediatric breast complaints in the emergency setting are comparable between subspecialties.

## Introduction

While pediatric breast cancer is rare, malignancy remains in the differential diagnosis of breast-related symptoms in this population, especially those patients presenting with a palpable breast mass [1, 2]. Currently, there are no specific breast cancer screening protocols for the pediatric population [3–5]. Therefore, breast-related symptoms in this population may alarm both the parent and child when present. Although most causes for these symptoms are benign, newly appearing symptoms, especially a new palpable mass, always require further medical evaluation as this can be an initial presenting symptom of a breast cancer [6, 7].

Pediatric patients with breast-related symptoms often initially present to the emergency department for evaluation. Unlike in the adult population, ultrasound is typically the initial imaging modality of choice for work-up instead of mammography due to the lack of ionizing radiation and higher sensitivity in dense breast tissue [8, 9]. Depending on the institution, pediatric breast ultrasound examinations performed in the emergency setting may be interpreted by either the pediatric or breast imaging division. While pediatric radiologists are accustomed to evaluating acute infectious and traumatic etiologies, they may be less familiar with breast-specific findings such as cluster of microcysts, complicated cysts, and invasive carcinomas which are predominantly evaluated by breast imaging radiologists. This can present a diagnostic challenge for pediatric radiologists if asked to interpret these studies and can lead to increased anxiety for radiologists, providers, pediatric patients, and their parents.

To optimize the breast care provided to pediatric patients in the emergency setting, adequate evaluation of breast complaints without missing suspicious lesions is vital for the initial work-up. Therefore, investigating the existence of discrepancies between management recommendations given by pediatric radiologists and breast imaging radiologists is relevant and can improve the quality of patient care. The purpose of this study is to compare management recommendations given by pediatric and breast imaging radiologists for pediatric breast ultrasounds performed in the emergency setting.

## Materials and methods

### Subjects and study design

This study has been reviewed and determined to be exempt by our institutional review board and Health Insurance Portability and Accountability Act (HIPAA) compliant.

This retrospective cohort study reviewed data from all pediatric breast ultrasounds performed in the emergency setting for patients ages 0 to 18 years from a single, multisite academic institution from 1/1/14 to 12/31/19. Our institution’s Value Analytics team then extracted patient medical record numbers (MRNs) from Epic which met our inclusion criteria. At our institution, these studies are interpreted by pediatric radiologists. There were 12 pediatric radiologists who interpreted pediatric breast ultrasounds during the study period with experience ranging from 1 to 33 years. Potential covariates were extracted from Epic including patient age at the time of the exam, race, and specific pediatric radiologist interpreting the examination. Pediatric breast ultrasound report impression and management recommendations, follow-up instructions given by the emergency department at discharge, and patient follow-up outcomes were recorded by manual chart review for each patient by one of the authors.

Three breast imaging radiologists (with 3, 8, and 25 years of experience) retrospectively reviewed each case independently and assigned a Breast Imaging Reporting and Data System (BI-RADS) category assessment. The breast imagers were only provided the patient’s age at the time of the exam and the indication for each ultrasound exam. They were not provided with the pediatric radiologist’s interpretation and recommendations and asked not to look at the report when reviewing each case.

Further management required for a case was defined as the recommendation by the pediatric radiologists for the patient to follow-up with the outpatient breast imaging clinic for further evaluation (*n* = 17) or a BI-RADS 3, 4, or 5 assessments given by the breast imaging radiologists (*n* = 18). A BI-RADS 3 assessment is given when a finding is considered probably benign (≤ 2% chance of malignancy) and is closely monitored using a standard surveillance imaging protocol at 6, 12, and 24 months after initial BI-RADS 3 assessment examination [10]. A BI-RADS 4 or 5 assessment is given when a finding is considered suspicious (> 2% but < 95% chance of malignancy or ≥ 95% chance of malignancy, respectively) and a biopsy is recommended for further evaluation [10].

The gold standard for each case (to which the pediatric radiologists were compared) was either patient clinical outcome, unanimous consensus among all three breast imaging radiologists, or the recommendation of the most experienced breast imaging radiologist when there was no consensus among all three breast imagers.

### Statistical analysis

All statistical analyses were performed using the computing software program SPSS, version 25 (2017 IBM Corp, Armonk, NY) and R (R Foundation for Statistical Computing, Vienna, Austria). The primary outcome of interest was differences in management recommendations between pediatric and breast imaging radiologists in the overall cohort and stratified between prepubertal (< 12 years of age) and pubertal age group. Differences in these recommendations were compared using Fisher’s exact test. Cohen’s kappa was used to assess agreement between subspecialty radiologists. Krippendorff’s alpha was used to assess agreement between the three breast imaging radiologists. Associations between post-discharge adherence rates and patient sociodemographic characteristics were evaluated using univariate and multivariate logistic regression. *p*-value ≤ 0.05 was considered statistically significant.

## Results

This study included 75 pediatric patients, with mean age 13 ± 5.6 years (Table 1). Table 2 shows the interpreted findings for all 75 cases by both the pediatric and the most experienced breast imaging radiologist. Pediatric radiologists and the most experienced breast imaging radiologist had moderate agreement in management recommendations (*k* = 0.54). Although the pediatric radiologists recommended follow-up more often than the most experienced breast radiologist, this difference was not statistically significant (22/75 [29.3%] vs 15/75 [20.0%], *p* = 0.26). This did not change when only looking at the pubertal (*N* = 61) group: (22/61 [36.1%] vs 15/61 [24.6%], *p* = 0.24). No further management recommendations were given in the prepubertal group by either the pediatric or breast radiologists.

**Table 1:** Pediatric emergency breast ultrasound study cohort characteristics

Characteristic	*N* = 75^*1*^
Age (years)	13 (5.6)
Race	
White	10 (13.3%)
Black	53 (70.1%)
Other	12 (16.0%)
US Indication	
Palpable lump	38 (50.7%)
Palpable lump and breast pain	19 (25.3%)
Breast pain	14 (18.7%)
Nipple discharge	2 (2.7%)
Breast swelling	2 (2.7%)
US findings	
Benign etiology	27 (36.0%)
Infectious etiology	34 (45.3%)
Mass	14 (18.7%)
Pediatric radiologist cohort	
Further management required	22 (29.3%)
No further management required	53 (70.7%)
Breast imaging radiologist A (3 years of experience)	
Further management required	22 (29.3%)
No further management required	53 (70.7%)
Breast imaging radiologist B (8 years of experience)	
Further management required	17 (22.7%)
No further management required	58 (77.3%)
Breast imaging radiologist C (25 years of experience)	
Further management required	15 (20.0%)
No further management required	60 (80.0%)

^*1 *^Mean (SD); *n* (%)

**Table 2:** Imaging findings and management recommendation comparison between pediatric radiologists and breast imager C (25 years of experience)

Case	Age at exam (years)	US indication	Pediatric radiologist cohort (management recommendation?)	Breast imaging radiologist C (BI-RADS 3/4 assessment?)
1	7.00	Palpable lump	Normal breast tissue	Normal breast tissue
2	12.00	Palpable lump	Abscess (FMR)^†^	Abscess (BI-RADS 3)
3	1.08	Breast swelling	Normal breast bud	Normal breast bud
4	0.50	Palpable lump	Phlegmon	Mastitis
5	15.00	Breast pain	Abscess	Abscess
6	18.00	Breast swelling	Abscess	Abscess
7	16.00	palpable lump	Normal breast tissue	Normal breast tissue
8	12.00	Palpable lump	Abscess	Abscess
9	17.00	Palpable lump and breast pain	Normal breast tissue	Normal breast tissue
10	18.00	Palpable lump	Sebaceous cyst	Sebaceous cyst
11	0.25	Palpable lump	Phlegmon	Mastitis
12	12.00	Palpable lump	Abscess	Abscess
13	17.00	Palpable lump	Cellulitis	Cellulitis
14	0.58	Palpable lump	Abscess	Abscess
15	14.00	Palpable lump and breast pain	Ductal ectasia (FMR) ^†^	Ductal ectasia
16	12.00	Palpable lump	Complicated cyst	Complicated cyst
17	0.67	Breast pain	Abscess	Abscess
18	18.00	Palpable lump	Mass (FMR)	Mass (BI-RADS 4)
19	15.00	Breast pain	Mastitis with ductal ectasia (FMR)	Mastitis with ductal ectasia
20	16.00	Palpable lump and breast pain	Mass	Mass (BI-RADS 4)
21	12.00	Palpable lump and breast pain	Inflamed cyst	Inflamed cyst
22	16.00	Palpable lump and breast pain	Cellulitis	Cellulitis
23	15.00	Palpable lump and breast pain	Mastitis with ductal ectasia	Mastitis
24	15.00	Palpable lump	Mass (FMR) ^†^	Mass (BI-RADS 4)
25	16.00	Palpable lump	Normal breast tissue	Normal breast tissue
26	18.00	Palpable lump and breast pain	Mastitis with ductal ectasia	Mastitis
27	12.00	Palpable lump	Normal breast tissue	Normal breast tissue
28	16.00	Palpable lump	Mass (FMR) ^†^	Mass (BI-RADS 4)
29	13.00	Breast pain	Complicated cyst	Complicated cyst
30	14.00	Palpable lump	Abscess	Abscess
31	16.00	Palpable lump	Mass	Mass (BI-RADS 4)
32	15.00	Palpable lump and breast pain	Complicated cyst (FMR) ^†^	Complicated cyst
33	14.00	Palpable lump	Abscess	Abscess
34	18.00	Breast pain	Mass (FMR) ^†^	Mass (BI-RADS 4)
35	16.00	Palpable lump and breast pain	Sebaceous cyst	Sebaceous cyst
36	15.00	Breast pain	Normal breast tissue	Normal breast tissue
37	17.00	Nipple discharge	Normal breast tissue	Normal breast tissue
38	0.08	Nipple discharge	Normal breast bud	Normal breast bud
39	18.00	Palpable lump	Mastitis with ductal ectasia (FMR) ^†^	Normal breast tissue
40	18.00	Palpable lump and breast pain	Inflamed sebaceous cyst (FMR) ^†^	Inflamed sebaceous cyst
41	18.00	Palpable lump and breast pain	Sebaceous cyst (FMR) ^†^	Sebaceous cyst
42	15.00	Palpable lump	Mass (FMR) ^†^	Mass (BI-RADS 4)
43	15.00	Palpable lump and breast pain	Mass	Mass (BI-RADS 4)
44	13.00	Palpable lump and breast pain	Mastitis	Mastitis
45	18.00	Palpable lump	Normal breast tissue	Normal breast tissue
46	17.00	Palpable lump and breast pain	Mass (FMR)	Mass (BI-RADS 4)
47	10.00	Breast pain	Normal breast tissue	Normal breast tissue
48	15.00	Palpable lump	Mass (FMR)	Mass (BI-RADS 4)
49	0.33	Palpable lump	Abscess	Abscess
50	12.00	Palpable lump	Abscess (FMR) ^†^	Abscess
51	18.00	Palpable lump	Abscess	Abscess
52	15.00	Palpable lump and breast pain	Mastitis with ductal ectasia (FMR) ^†^	Mastitis with ductal ectasia
53	13.00	Breast pain	Normal breast tissue	Normal breast tissue
54	0.08	Palpable lump	Normal breast tissue	Breast edema
55	5.00	Palpable lump	Cellulitis	Cellulitis
56	13.00	Palpable lump	Abscess	Abscess
57	17.00	Palpable lump	Abscess	Abscess
58	14.00	Breast pain	Simple cyst	Simple cyst
59	16.00	Breast pain	Mastitis	Mastitis
60	0.67	Palpable lump	Normal breast bud	Normal breast bud
61	18.00	Breast pain	Cellulitis	Breast edema
62	0.08	Palpable lump	Mastitis	Breast edema
63	9.00	Palpable lump	Mastitis	Mastitis
64	18.00	Palpable lump	Mass (FMR) ^†^	Mass (BI-RADS 4)
65*	17.00	Palpable lump	Mass (FMR)	Mass (BI-RADS 4)
66	17.00	Breast pain	Normal breast tissue	Normal breast tissue
67	16.00	Palpable lump	Abscess (FMR)	Abscess
68	12.00	Breast pain	Ductal ectasia (FMR)	Normal breast tissue
69	17.00	Palpable lump	Normal breast tissue	Normal breast tissue
70	14.00	Palpable lump and breast pain	Abscess	Abscess
71	15.00	Breast pain	Abscess	Abscess
72	15.00	Palpable lump and breast pain	Abscess	Abscess
73	14.00	Palpable lump	Mass (FMR) ^†^	Mass (BI-RADS 4)
74	17.00	Palpable lump and breast pain	Mastitis	Mastitis
75	14.00	Palpable lump and breast pain	Mass (FMR) ^†^	Mass (BI-RADS 4)

^*^Malignant case

^†^Patient lost to follow-up

*FMR*, further management required

Of the 13 cases which had discrepant management recommendations between the pediatric and breast radiologists, 7 (53.8%) cases were lost to follow-up (Table 2: patients 15, 32, 39, 40, 41, 50, and 52), in 3 (23.1%) cases, no follow-up recommendations were given by the pediatric radiologists (Table 2 patients 20, 31, and 43) and had subsequent follow-up in the medical record upon chart review, and 3 (23.1%) cases had patient follow-up with US interpretation matching those of breast imaging radiologist C (Table 2: patients 19, 67, and 68).

Breast imaging radiologists had near perfect agreement in management recommendations (*k* = 0.81). Of the 14 masses interpreted as suspicious by all three breast imagers (all in the pubertal age group), there was no significant difference in recommendations for further management between the breast radiologists and the pediatric radiologists (14/14 [100%] vs 11/14 [78.6%] respectively, *p* = 0.22). Of the 11 masses with further management recommendations by the pediatric radiologists, 7 (63.6%) patients were lost to follow-up and the remaining 4 (36.3%) patients underwent biopsy: 3 were fibroadenoma and 1 was malignant spindle cell tumor. All 3 masses which had discrepant management recommendations between the pediatric and breast radiologists (Table 2: patients 20, 31, and 43) were described as “oval, circumscribed mass likely representing a fibroadenoma” as described on the report by the pediatric radiologists at the time of US interpretation. In these 3 cases, no follow-up was recommended by the pediatric radiologists whereas the breast imaging radiologists assigned these cases with a BI-RADS 4.

**Fig. 1:**
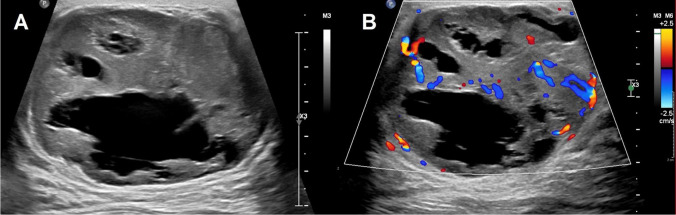
Breast ultrasound with indication of a palpable lump in a 17-year-old female showed a complex cystic and solid mass with circumscribed margins with internal vascularity in the left breast at 7:00 position, 5 cm from the nipple. Ultrasound image (**A)** shows a complex cystic and solid mass with circumscribed margins at the 7:00 position, 5 cm from the nipple, measuring 6.2 × 5.3 × 6.7 cm with internal vascularity (**B**). This finding was recommended for further evaluation at the breast clinic by the pediatric radiologist. Finding was given a BI-RADS 4 assessment by the breast imaging radiologist at the follow-up examination and subsequently biopsied resulting in malignant spindle cell tumor

All patients with malignant outcomes (1/75 [1.3%]) were given the appropriate initial recommendation to be further evaluated at the breast clinic by the pediatric radiologists (Fig. 1).

The majority of patients who were recommended to have further management with the breast clinic by pediatric radiologists (68.2% [15/22]) were lost to follow-up. While the majority of patients lost to follow-up were of Black race (86.7% [13/15]), no sociodemographic factor was significantly associated with non-adherence to follow-up recommendations on univariate analysis (Table 3).

**Table 3:** Univariate logistic regression analysis for adherence to follow-up recommendations after discharge from the emergency department

Characteristic	Univariate analysis			
	*N*	OR^*1*^	95% CI^*1*^	*p*-value
Age	22	1.06	0.68, 1.73	0.79
Race	22			
White		—	—	
Black		0.38	0.01, 11.0	0.53
Other		1.00	0.01, 69.5	> 0.99

## Discussion

Our study demonstrated that there is no clinically significant difference in management recommendations for pediatric breast ultrasound examinations in the emergency setting between pediatric and breast imaging radiologists. However, the majority of patients were non-adherent to follow-up recommendations by the pediatric radiologists after being discharged from the emergency department.

Although the majority of pediatric breasts complaints are related to benign etiologies, the risk of malignancy is not negligible, especially when the presenting complaint is a palpable lump [1, 6, 7]. The types of malignant breast pathologies encountered in the pediatric setting are phyllodes tumor, primary breast cancer (secretory adenocarcinoma, medullary, and inflammatory), and metastatic disease [11]. The initial imaging evaluation of pediatric breast complaints is often performed in the emergency setting and interpreted by pediatric radiologists, not breast imaging radiologists. Elective rotations and didactic lectures in breast imaging are not commonly included in pediatric radiology fellowship curriculums [12–15]. Thus, it is understandable that pediatric radiologists may be less familiar with breast-specific findings, as the only exposure is through the emergency setting, where the work-up is usually incomplete. Our study demonstrated that despite these limitations, no malignancies were misdiagnosed as benign in the emergency setting when interpreted by pediatric radiologists. Clinically, while the pediatric radiologists recommended more follow-up for findings that were interpreted as benign by the most experienced breast imaging radiologist, there was no statistical difference in management recommendations between subspecialty radiologists. Therefore, the initial evaluation of acute pediatric breast complaints can be adequately performed in the emergency department without missing suspicious findings that require further evaluation in the outpatient breast clinic.

Our study had three masses which had discrepant management recommendations between the pediatric and breast radiologists. Per the ultrasound report, all three masses were described by the pediatric radiologists as being oval, circumscribed masses which likely represented fibroadenomas. The discrepancy in management recommendations may be related to the differences in how pediatric and breast imaging radiologists practice. Breast imaging radiologists’ practice patterns and management must follow the BI-RADS Atlas whereas pediatric radiologists who are interpreting breast examinations do not [10]. Breast imaging radiologists are more cautious as the BI-RADS Atlas states that biopsy recommendations are given for a finding which has over a 2% chance of malignancy [10]. On the other hand, pediatric radiologists’ recommendations are not based on stringent statistics, but on interpreting experience. In the pediatric population, fibroadenomas account for 68% of all breast masses and up to 94% of biopsied breast masses in this population [16]. Recommendations when the imaging findings are classic for fibroadenoma are not standardized among pediatric radiologists as they are among breast imaging radiologists. Some pediatric radiologists would recommend follow-up with the breast imaging clinic in this situation, while others would not recommend any follow-up. Therefore, while there was a difference in management recommendations between the two subspecialties, the interpretation of the mass was likely not different nor the final pathologic outcome of the mass. Moving forward, more communication between the two subspecialties may be needed to better standardize recommendations for referring providers and patients.

Prior literature has demonstrated that adherence to follow-up recommendations after discharge from the pediatric emergency department is poor [17, 18]. Our study supports this, as majority of our study cohort patients who were recommended for imaging follow-up did not adhere to these recommendations after discharge. Our study also supports the findings of Wang et al. that race was not significantly associated with non-adherence to follow-up recommendations [18]. All children, regardless of sociodemographic characteristics, are at risk for poor adherence and interventions aimed at improving patient follow up after discharge are necessary. Curran et al. found that improving discharge communication instructions increased not only patient comprehension, but also overall health outcomes [19]. Patient navigators, commonly utilized in breast imaging to improve patient adherence to screening mammography guidelines and diagnostic mammography follow-up, have also been shown to improve outpatient follow-up adherence after discharge from the pediatric emergency department [20, 21]. Therefore, for follow-up recommendations by pediatric radiologists to be impactful for clinical outcomes, adherence to these recommendations is vital and incorporation of post-discharge interventions is needed to prevent delayed or missed diagnosis of pediatric breast cancer.

Our study has a few limitations. This study was performed at a single, multisite academic institution and our small sample size could have affected the generalizability of our results. Larger sample size and multisite analysis may be warranted to confirm our findings and expose any disparities which may be associated with follow-up adherence rates. Additionally, we are unable to capture those patients who may have had follow-up performed at a different institution therefore patient non-adherence to follow-up recommendations is another limitation. Our malignancy rate may have been underestimated due to low adherence follow-up rates among patients with masses; however, given the very low incidence of breast cancer in the pediatric population, this likely would not have changed the overall conclusion of our study.

In conclusion, management recommendations for pediatric breast complaints in the emergency setting are comparable between pediatric and breast radiologists. Initial evaluation of pediatric breast complaints can be adequately interpreted by pediatric radiologists in the emergency setting without missing suspicious lesions. However, there is a deficit in consistent follow-up of pediatric breast-specific findings initially evaluated in the emergency setting; therefore, more frequent and targeted interventions to promote follow-up adherence are required post-discharge from the emergency department to reduce the likelihood of a missed or delayed cancer diagnosis.
